# Yield-Enhancing Heterotic QTL Transferred from Wild Species to Cultivated Rice *Oryza sativa* L

**DOI:** 10.1371/journal.pone.0096939

**Published:** 2014-06-20

**Authors:** Kiran B. Gaikwad, Naveen Singh, Dharminder Bhatia, Rupinder Kaur, Navtej S. Bains, Tajinder S. Bharaj, Kuldeep Singh

**Affiliations:** 1 Department of Plant Breeding and Genetics, Punjab Agricultural University, Ludhiana, India; 2 School of Agricultural Biotechnology, Punjab Agricultural University, Ludhiana, India; International Rice Research Institute, Philippines

## Abstract

Utilization of “hidden genes” from wild species has emerged as a novel option for enrichment of genetic diversity for productivity traits. In rice we have generated more than 2000 lines having introgression from ‘A’ genome-donor wild species of rice in the genetic background of popular varieties PR114 and Pusa44 were developed. Out of these, based on agronomic acceptability, 318 lines were used for developing rice hybrids to assess the effect of introgressions in heterozygous state. These introgression lines and their recurrent parents, possessing fertility restoration ability for wild abortive (WA) cytoplasm, were crossed with cytoplasmic male sterile (CMS) line PMS17A to develop hybrids. Hybrids developed from recurrent parents were used as checks to compare the performance of 318 hybrids developed by hybridizing alien introgression lines with PMS17A. Seventeen hybrids expressed a significant increase in yield and its component traits over check hybrids. These 17 hybrids were re-evaluated in large-size replicated plots. Of these, four hybrids, viz., ILH299, ILH326, ILH867 and ILH901, having introgressions from *O. rufipogon* and two hybrids (ILH921 and ILH951) having introgressions from *O. nivara* showed significant heterosis over parental introgression line, recurrent parents and check hybrids for grain yield-related traits. Alien introgressions were detected in the lines taken as male parents for developing six superior hybrids, using a set of 100 polymorphic simple sequence repeat (SSR) markers. Percent introgression showed a range of 2.24 from in *O. nivara* to 7.66 from *O. rufipogon*. The introgressed regions and their putative association with yield components in hybrids is reported and discussed.

## Introduction

Rice is grown annually on more than 153 million hectares with an annual production of about 483 million tons globally [1]. Remarkable progress has been achieved in rice production and productivity during the past 50 years, but still, food security is among the biggest challenges of the 21st century. Global rice demand is estimated to rise from 676 million tons in 2010 to 763 million tons in 2020 and further increase to 852 million tons in 2035. This is an overall increase of 26% or 176 million tons in 25 years [2]. Rice productivity witnessed a tremendous increase during the green revolution phase (late 1960s to 1970s); however, these gains slowly reached a plateau. Hybrid-rice technology is advocated as a viable option for improving productivity, as has been amply demonstrated in Peoples' Republic of China [3]. Tremendous genetic variability exists in cultivated rice, but this is just five percent of the total variability existing in the genus *Oryza* [4]. Plant breeders have recognized the worth of wild species and used these for improving simply inherited traits. The most successful examples of utilizing wild species include the use of *O. spontanea* as a source of wild abortive cytoplasmic male sterility [5], *O. nivara* genes to provide resistance against grassy stunt virus [6], and *O. longistaminata* gene *Xa21* [7]–[8], *O. rufipogon* gene *Xa23* [9] and *O. nivara* gene *Xa38* [10] for resistance against bacterial blight. There is growing concern that limited genetic diversity in present day high-yielding rice germplasm impedes further improvement in productivity. Incorporation of potentially favorable alleles from wild ancestors of rice into improved genotypes for productivity traits has emerged as a promising strategy. The foundation of utilizing productivity-enhancing genes from wild species was laid by Frey et al. [11] in oats. Following the availability of advanced-backcross quantitative trait loci (AB-QTL) approach proposed by Tanksley and Nelson [12], discovery of favorable genes from wild species received impetus as a plant breeding strategy. Consequently, several yield-enhancing alleles from wild species of rice, such as *O. rufipogon*, were identified and introduced into high-yielding elite cultivars [13]–[20]. Apart from *O. rufipogon*, search for yield-enhancing quantitative trait loci (QTL) alleles was successfully extended to *O. glaberrima* [21], *O. grandiglumis* [22], *O. glumaepatula* [23], *O. longistaminata* [24] and *O. minuta* [25]. The lines generated provided precise estimates of genetic effects of introgression under relatively uniform and elite genetic backgrounds [12]. Furthermore, there are several indications that introgression lines served as dynamic entities to identify new genes [26], distinguish pleiotropy from linkage [27] and identify QTL contributing to heterosis, particularly those showing overdominance [28].

In rice, superiority of introgression lines over recurrent parents has been reported by many workers, but the effect of alien introgression in F_1_ hybrids has not been studied extensively. This study was conceived with the hypothesis that alien introgression(s) in homozygous condition may not be able to contribute positively to the phenotype because of replacement of a cultivated genome segment. However, in heterozygous condition, the recipient complement is conserved and minor negative effect associated with the alien segment is likely to be masked. Therefore, the present study was planned to determine the effect of novel genes from wild species on the yielding ability of hybrids and to locate the alien segments responsible for enhanced magnitude of heterosis in rice hybrids.

## Materials and Methods

### Development of introgression lines by backcross breeding

**Figure 1:**
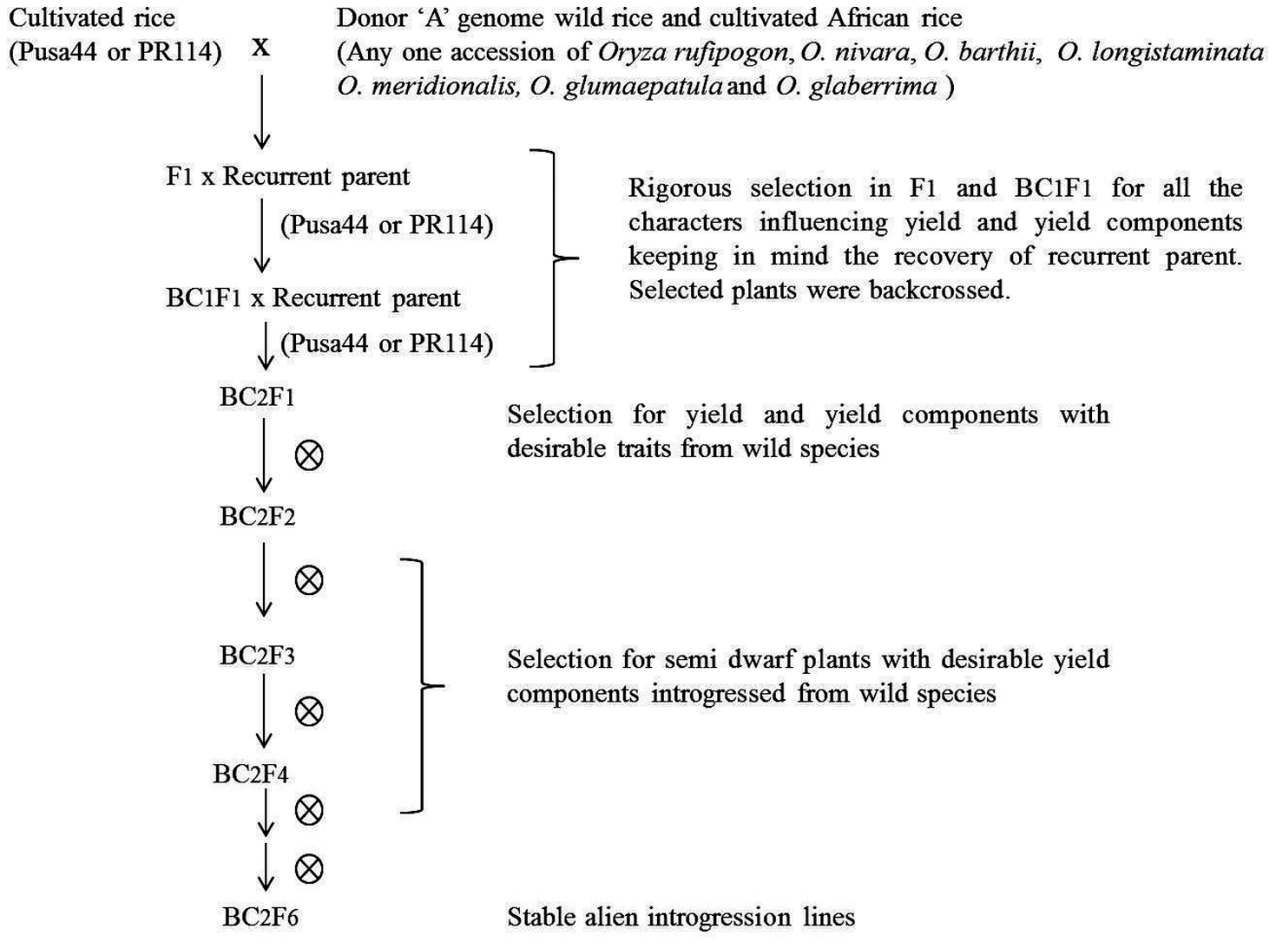
Development of alien introgression lines.

A backcross breeding programme was initiated to introgress useful variability for improving yield and yield components from wild *Oryza* species into two high yielding indica rice varieties, viz., Pusa44 and PR114. These varieties are widely adapted and restore fertility in wild abortive (WA) cytoplasm from *O. spontanea*. Forty-five accessions of various ‘A’ genome wild species, viz., *O. rufipogon*, *O. nivara*, *O. barthii*, *O. longistaminata*, *O. glumaepatula*, *O. meridionalis* and cultivated African rice *O. glaberrima* were used as donors. Two to three backcrosses, followed by continuous selfing for five or more generations, led to the development of >2000 alien introgression lines (ILs). During backcrossing and selfing, phenotypic selection was practiced for yield, yield component traits and phenotypic acceptability (Fig. 1).

### Development and evaluation of hybrids

**Figure 2:**
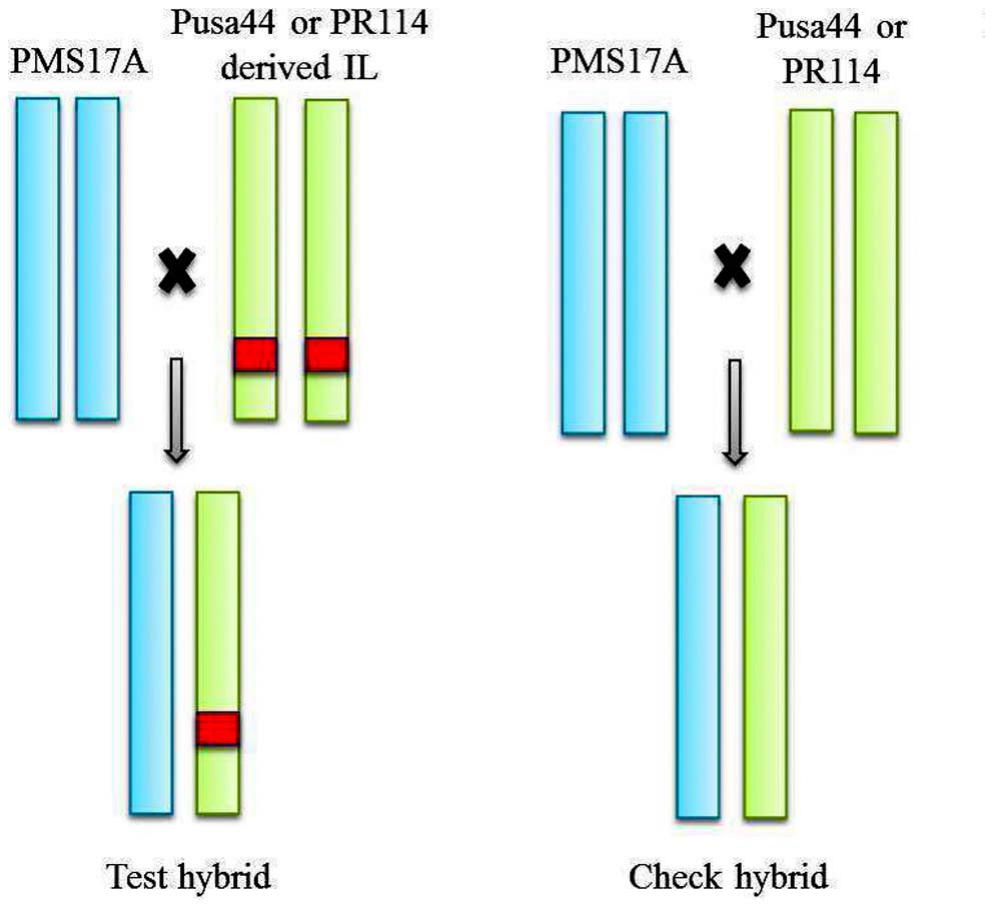
Development of Test and Check hybrids.

Based on agronomic evaluation of a large set of introgression lines in an earlier experiment, 318 ILs were selected and crossed with a CMS line PMS17A, carrying ‘WA’ cytoplasm to develop test hybrids. Among the 318 ILs used for generating test hybrids, 99 were derived from *O. rufipogon*, 99 from *O. nivara*, 79 from *O. glaberrima*, 30 from O. *longistaminata*, six from O. *glumaepatula*, five from *O. barthii* and two from *O. meridionalis*, which were used as donors. Recurrent parents, viz., Pusa44 and PR114 were also crossed with the same CMS line to develop check hybrids. Both test and check hybrids had similar genetic background except for the alien introgression segments transferred from wild species (Fig. 2).

In 2008, test hybrids, check hybrids and recurrent parents were evaluated at Punjab Agricultural University Ludhiana, India for yield and yield-contributing traits in a simple lattice design with two replications and plot size of 1.26 m^2^. Observations were recorded on days to 50% flowering, days to maturity, plant height (cm), percent pollen fertility, panicle length (cm), spikelets per panicle, grains per panicle, percent spikelet fertility, 1000-grain weight (g) and grain yield (t/ha) following the standard evaluation system adopted at IRRI [29]. Based on plot yield and other contributing traits, 17 promising hybrids were identified. F_1_ seeds of the 17 promising test hybrids and two check hybrids, viz., PMS17A/Pusa44 and PMS17A/PR114, were generated at Central Rice Research Institute (CRRI), Cuttack, India during winter season 2008–2009 for further evaluation. In 2009, the 17 test hybrids along with hybrid checks and recurrent parents were evaluated at Ludhiana in a randomized block design (RBD) using a plot size of 7.02 m^2^ for each test entry. This evaluation was carried out in two experiments. Experiment I was planned to evaluate four test hybrids developed from Pusa44-derived ILs and 13 test hybrids developed from PR114-derived ILs were evaluated in experiment II. Statistical analyses were performed on the basis of entry means in each replication. Analysis of variance for simple lattice and randomized block design was carried out using software SPAR 2.0 [30].

### Marker analysis of introgression lines

DNA from the ILs, recurrent and donor parent lines was isolated from young leaves following CTAB (Cetyl Trimethyl Ammonium Bromide) method as modified by Saghai-Maroof et al. [31] and analyzed by using SSR markers [32]–[33]. Markers spanning the whole genome at approximately 10 cM or closer intervals were used to assess parental polymorphism among the donor and the recipient line using 2.5% agarose gel. Out of 200 SSR markers screened, 100 markers were polymorphic. To precisely estimate the size of alien chromosome segments, additional markers were screened in the regions harboring these introgressions. These polymorphic markers were used for analyzing the introgression and generating graphical genotypes of each introgression line using the software GGT 2.0 [34].

## Results

### Agronomic performance of introgression line hybrids (ILHs)

Analysis of variance revealed that mean squares attributable to genotypes were highly significant for all the traits, indicating considerable amount of genetic variation in the material used in this study (Table S1, Figure S1 a–f). Out of the 318 test hybrids evaluated, some recorded a significant increase in yield components (Figure S1, a–f) while others, especially ones that had introgressions from *O. glaberrima* showed 100% spikelet sterility in some cases. Based on better yielding ability and phenotypic performance over the check hybrids, 17 hybrids were identified. These hybrids and their respective check hybrids had similar genetic background except for introgressed segments. Mean grain yield and percent standard heterosis of superior introgression line hybrids over their respective check hybrids are presented in Table 1. Four hybrids, viz., ILH299, ILH326, ILH411 and ILH435, were having Pusa44 with introgression enriched genome as male parent showed significantly higher yield over check hybrid PMS17A/Pusa44. This increase in standard heterosis ranged from 37% to 40% over the check hybrid. ILH299 and ILH326 carried introgressions from *O. rufipogon*, whereas ILH411 and ILH435 had introgressions from *O. nivara*. In the second set of 13 hybrids, having enriched genome of PR114, 10 had significant positive heterosis (22.6 to 44.3%) for grain yield over check hybrid PMS17A/PR114. ILH867 (PMS17A/IL867) having introgressions from *O. rufipogon* (Acc. IRGC104433) showed highest heterosis of 44.32%. These 17 hybrids were also superior over their respective recurrent parents, viz., Pusa44 and PR114, for yield and yield-contributing traits under study (data not shown). From this initial evaluation, it was clear that introgression segments from wild species in hybrid background contributed positively for yield improvement.

**Table 1:** Mean grain yield and standard heterosis of 17 ILHs evaluated in the year 2008.

Hybrid ID	Cross	Grain yield (t/ha)	Percent heterosis over respective check hybrids
ILH299	PMS17A/IL299 [Pusa44/*O. rufipogon* IRGC100219//2*Pusa44]	7.691	37.1**
ILH326	PMS17A/IL326 [Pusa44/*O. rufipogon* IRGC100219//2*Pusa44]	7.595	35.4**
ILH411	PMS17A/IL411 [Pusa44/*O. nivara* (CR100113A//2*Pusa44]	7.637	36.2**
ILH435	PMS17A/IL435 [Pusa44/*O. nivara* (CR100142A//2*Pusa44]	7.829	39.6**
ILH867	PMS17A/IL867 [PR114/*O. rufipogon* IRGC104433//2*PR114]	8.906	44.3**
ILH873	PMS17A/IL873 [PR114/*O. rufipogon* IRGC104433//2*PR114]	7.254	26.2*
ILH984	PMS17A/IL894 [PR114/*O. rufipogon* IRGC104433//2*PR114]	7.375	19.5
ILH896	PMS17A/IL896 [PR114/*O. rufipogon* IRGC104433//2*PR114]	7.702	24.8*
ILH900	PMS17A/IL900 [PR114/*O. rufipogon* IRGC104433//2*PR114]	7.568	22.6*
ILH901	PMS17A/IL901 [PR114/*O. rufipogon* IRGC104433//2*PR114]	8.102	32.6**
ILH921	PMS17A/IL921 [PR114/*O. nivara* IRGC100593//2*PR114]	8.097	31.2**
ILH924	PMS17A/IL924 [PR114/*O. nivara* IRGC105695//2*PR114]	7.377	19.5
ILH929	PMS17A/IL929 [PR114/*O. nivara* IRGC100114//2*PR114]	7.977	29.2**
ILH951	PMS17A/IL951 [PR114/*O. nivara* (CR100142A//2*PR114]	7.810	19.1
ILH1011	PMS17A/IL1011 [PR114/*O. nivara* (CR100140//2*PR114]	8.391	35.9**
ILH1459	PMS17A/IL1459 [PR114/*O. glumaepatula* (IR104387//2*PR114]	8.034	36.96**
ILH1433	PMS17A/IL1433 [PR114/*O. longistaminata* (IR104301B//2*PR114]	7.558	23.35*
Check Hybrid 1	PMS17A/Pusa44	5.627	-
Check Hybrid 2	PMS17A/PR114	6.127	-

*, **significant at P≤0.05 and P≤0.01, respectively. IRGC and CR refer to International Rice Germplasm Centre and CRRI Cuttack, accession numbers, respectively.

In 2009, the 17 hybrids were again evaluated, along with their parents, including introgression lines and PMS17B (maintainer of PMS17A), two check hybrids, viz., PMS17A/Pusa44 and PMS17A/PR114, and recurrent parents (Pusa44 and PR114) for yield and yield-contributing traits. Analysis of variance revealed significant genotypic differences for all the traits except productive tillers per plant (Table S1). To assess the effect of alien introgression segments in homozygous state, the introgression lines were compared with their recurrent parents, i.e., Pusa44 and PR114, for important yield-contributing traits (Table 2). Introgression lines, viz., IL299, IL326, IL411 and IL435, showed lower yield, whereas IL326 showed significantly fewer spikelets per panicle and grains per panicle than their recurrent parent Pusa44. Out of 13 introgression lines in PR114 genetic background, seven lines had marginal but non-significant increase in yield over PR114. IL867, IL894, IL921 and IL1459 showed significant increase for spikelets per panicle over PR114; however, they failed to show more grains per panicle than its recurrent parent because of lower spikelet fertility. None of introgression lines showed any improvement in grains per panicle and 1000-grain weight over PR114. Results indicated that most of these introgression lines failed to surpass their recurrent parents for important yield-contributing traits.

**Table 2:** Performance of 17 introgression lines for important yield contributing traits.

Introgression Line	Generation	Panicle length (cm)	Spikelets per panicle	Grains per panicle	1000 grain weight (g)	Grain Yield (t/ha)	Percent yield increase/decrease over Pusa44 or PR114
IL299	BC_2_F_6_ [Pusa44/*O. rufipogon* IRGC100219//2*Pusa44]	25.42	172.77	124.97	21.73^c^	6.903	−13.95
IL326	BC_2_F_6_ [Pusa44/*O. rufipogon* IRGC100219//2*Pusa44]	23.67	111.07^c^	72.63^c^	23.26^c^	5.026^c^	−37.35
IL411	BC_2_F_8_ [Pusa44/*O. nivara* (CR100113A//2*Pusa44]	25.57	171.00	118.43^d^	22.74^c^	6.481	−19.21
IL435	BC_2_F_8_ [Pusa44/*O. nivara* (CR100142A//2*Pusa44]	23.52	168.00	115.00^d^	22.43^c^	6.470	−19.34
Pusa44	-	24.92	164.20	136.47	24.27	8.022	-
LSD (P≤0.05)		2.47	39.38	25.36	1.41	2.407	-
LSD (P≤0.01)		1.70	27.08	17.44	0.97	1.655	-
IL867	BC_2_F_6_ [PR114/*O. rufipogon* IRGC104433//2*PR114]	25.26	181.70^a^	148.70	24.56^c^	7.759	−3.02
IL873	BC_2_F_6_ [PR114/*O. rufipogon* IRGC104433//2*PR114]	25.93^a^	145.10	130.93	23.47^c^	7.965	−0.46
IL894	BC_2_F_6_ [PR114/*O. rufipogon* IRGC104433//2*PR114]	24.00	174.47^b^	155.87	23.48^c^	8.388	4.84
IL896	BC_2_F_6_ [PR114/*O. rufipogon* IRGC104433//2*PR114]	25.14	152.50	132.53	26.91	8.992	12.39
IL900	BC_2_F_6_ [PR114/*O. rufipogon* IRGC 104433//2*PR114]	24.54	160.03	126.20^d^	26.97	9.046	16.81
IL901	BC_2_F_6_ [PR114/*O. rufipogon* IRGC104433//2*PR114]	25.02	149.87	130.90	25.89	8.742	9.27
IL921	BC_2_F_8_ [PR114/*O. nivara* IRGC100593//2*PR114]	30.27^a^	180.27^a^	130.77	22.85^c^	7.633	−4.60
IL924	BC_2_F_8_ [PR114/*O. nivara* IRGC105695//2*PR114]	24.63	145.40	124.30^d^	26.45	8.838	10.46
IL929	BC_2_F_8_ [PR114/*O. nivara* IRGC100114//2*PR114]	24.66	167.00	136.77	25.03^d^	7.991	−0.13
IL951	BC_2_F_8_ [PR114/*O. nivara* (CR100142A//2*PR114]	25.24	168.87	136.13	22.23	6.4.80^d^	−19.01
IL1011	BC_2_F_6_ [PR114/*O. nivara* (CR100140//2*PR114]	25.07	147.10	128.70	25.17	8.381	4.75
IL1459	BC_2_F_6_ [PR114/*O. glumaepatula* IRGC104387//2*PR114]	22.65^c^	188.20^a^	131.30	23.47^c^	6.597^d^	−17.54
IL1433	BC_2_F_6_ [PR114/*O. longistaminata* IRGC104301B//2*PR114]	26.66^a^	160.27	134.67	26.05	8.289	3.59
PR114	-	24.47	156.57	142.73	26.58	8.001	-
LSD (P≤0.05)		1.44	22.52	20.68	1.88	1.787	-
LSD (P≤0.01)		1.06	16.69	15.32	1.39	1.324	-

^a^ Significantly higher and ^c^ lower than the recurrent parent Pusa 44 or PR114 at *P*≤0.01.

^b^ Significantly higher and ^d^ lower than the recurrent parent Pusa 44 or PR114 at *P*≤0.05.

**Figure 3:**
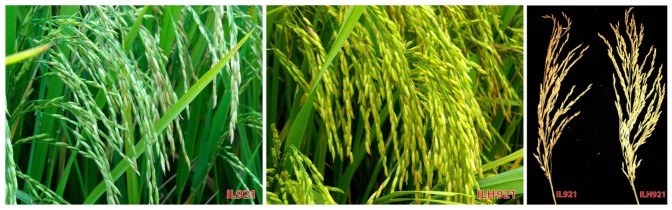
Field photograph of IL921 (left), ILH921 (middle) and on right hand side showing panicle characteristics.

To assess the effect of introgression in heterozygous condition, the 17 ILs were crossed with CMS line PMS17A. The extent of heterosis in these hybrids for important agronomic traits over the parents, check hybrids and recurrent parents of introgressions lines is presented in Table 3. Heterosis for grain yield is attributable to simultaneous manifestation of heterosis for various yield components. Out of the four hybrids, having Pusa44-derived introgression lines as one of the parent, ILH299 and ILH326 recorded significant positive heterosis to the tune of 30.4% and 74.3%, respectively, over their respective parental introgression lines IL299 and IL326. Both these hybrids recorded significant standard heterosis (>30%) over the check hybrid PMS17A/Pusa44. Remaining two hybrids, viz., ILH411 and ILH435, failed to show any improvement over parents and checks for grain yield. Out of 13 hybrids, having PR114-derived introgression lines as one of the parent, five, viz., ILH867, ILH901, ILH921, ILH951 and ILH1011, recorded significant positive heterosis over check hybrid PMS17A/PR114. ILH951 (PMS17A/IL951) recorded highest magnitude of heterosis for grain yield over IL951 (51.6%), PMS17A/PR114 (43.0%), PMS17B (18.4%) and PR114 (22.8%). Except for ILH1011, the remaining four hybrids showed a significant increase in grain yield over PR114. No improvement in yield was observed because of low grains per panicle and low spikelet fertility in rest of eight hybrids. Five hybrids, viz., ILH299, ILH326, ILH867, ILH921 and ILH951, which gave higher grain yield than introgression line parent and check hybrids, were also seen to possess higher grain weight. ILH299, ILH326, ILH86 and ILH901 carried introgressions from *O. rufipogon*, whereas ILH921 and ILH951 had introgressions from *O. nivara*. Yield increase in these six hybrids was not associated with delayed flowering or maturity. In fact, almost all hybrids were earlier in flowering than the check hybrids, introgression lines and the recurrent parents by 3 to 5 days (Table S2). Two hybrids (ILH867 and ILH921) were taller than check hybrids and PR114 but shorter than respective parental introgression lines IL867 and IL921. Remaining hybrids did not show any change in plant height. Thus, introgression for yield was not associated with change in plant architecture and prolonged crop duration in this study (Table S2). Genetic variation for productive tillers per plant was non-significant. However, for other yield-contributing traits, viz., panicle length and spikelets per panicle, ILH299, ILH326, ILH867, ILH873, ILH901, ILH921 and ILH951 showed significant improvement over introgressions lines, check hybrids and respective recurrent parents. For panicle length, ILH921 (PMS17A/IL921) recorded 23% heterosis over male parent, whereas ILH951 (PMS17A/IL951) recorded 43.3% and 54.5% heterosis over IL951 and PR114, respectively. ILH921 had highest panicle length among all the hybrids (Fig. 3). For grains per panicle, four hybrids, viz., ILH299, ILH326, ILH921 and ILH951, recorded significant positive heterosis (>30%) over their respective introgression lines, check hybrid PMS17A/Pusa44 (20–35%) and Pusa44 (24–38%). In 2008, out of 318 hybrids tested, 60 hybrids having introgressions from *O. glaberrima* showed almost 100% spikelet sterility. Similarly, in 2009, almost all hybrids had lower spikelet fertility than recurrent parents except for hybrid ILH921, which expressed >10% significant heterosis over IL921, PMS17B and PMS17A/PR114. Spikelet sterility was inversely related to yield in hybrids. These hybrids were also tested for pollen fertility. High yielding hybrids, viz., ILH299, ILH326, ILH921 and ILH951, had higher pollen fertility than the check hybrids. From these results, it can be seen that introgressed segments in homozygous state failed to show improvement in grain yield in the introgression lines.

**Table 3:** Mean values of the ILHs for important agronomic traits and extent of heterosis (%) over parents check hybrids and recurrent parents in 17 hybrids.

Traits	Parameters	ILHs																
		ILH 299∧ a	ILH 326∧	ILH 411∧	ILH 435∧	ILH 867∼	ILH 873∼	ILH 894∼	ILH 896∼	ILH 900∼	ILH 901∼	ILH 921∼	ILH 924∼	ILH 929∼	ILH 951∼	ILH 1011∼	ILH 1459∼	ILH 1433∼
Panicle length (cm)	Mean of test hybrid	27.4	27.8	26.3	25.8	26.	27.6	24.8	25.5	25.3	26.9	29.4	25.2	25.0	27.7	27.6	25.3	23.7
	Heterosis over IL	**7.8** **	**17.6** **	2.9	**9.7** **	4.3	**6.5** *	3.5	1.6	3.4	**7.63** **	**23.8** **	2.5	1.6	**10.1** **	**10.3** **	**11.8** **	**−**10.9**
	Heterosis over PMS17B	**17.1** **	**18.9** **	**12.5** **	**10.3** **	**10.9** **	**16.2** **	4.5	**7.5** *	**6.8** *	**13.6** **	**23.8** **	**6.31** *	5.53	**16.9** **	**16.4** **	**6.5** *	−0.1
	Heterosis over CH 1or CH2	**9.3** **	**11.0** **	**5.0** *	2.9	**5.6** *	**10.8** **	−0.3	2.4	1.7	**8.0** **	**17.9** **	1.3	0.5	**11.4** **	**10.9** **	1.5	−4.8
	Heterosis over recurrent parent	**9.9** **	**11.7** **	**5.6** *	3.6	**7.7** **	**12.9** **	1.5	4.3	3.6	**10.0** **	**20.2** **	3.2	2.4	**13.5** **	**13.0** **	3.4	−3.0
Grains per panicle	Mean of test hybrid	175.3	173.4	130.0	157.2	172.6	140.5	150.0	123.9	124.7	168.2	180.0	115.2	114.7	181.4	158.0	157.0	138.8
	Heterosis over IL	**40.3** **	**38.8** **	2.4	**18.2** **	**16.0** **	7.3	−3.7	−6.4	−1.1	**28.5** **	**37.7** **	−7.3	−16.0**	**33.2** **	**22.8** **	**19.5** **	3.1
	Heterosis over PMS17B	**17.2** **	**15.9** **	−13.0**	5.0	9.0	−11.2*	−5.2	−21.7**	−21.1**	6.2	**13.7** **	−27.2**	−27.5**	**14.5** **	−0.1	−0.8	−12.2*
	Heterosis over CH 1or CH2	**35.6** **	**34.1** **	0.6	**21.6** **	**16.8** **	−4.8	1.5	−16.0**	−15.5**	**13.9** **	**21.9** **	−22.0**	−22.3**	**22.8** **	7.0	6.3	−5.9
	Heterosis over recurrent parent	**38.6** **	**37.1** **	2.8	**24.3** **	**20.9** **	−1.5	5.1	−13.1*	−12.5*	**17.8** **	**26.1** **	−19.2**	−19.5**	**27.0** **	**10.7** *	10.0	−2.7
Spikelet fertility (%)	Mean of test hybrid	84.4	83.2	64.5	71.8	78.2	66.9	72.5	63.5	62.3	75.5	80.3	56.4	59.1	85.4	74.9	73.8	57.6
	Heterosis over IL	2.5	**13.2** *	−9.5	−9.6*	−4.4	−25.8**	−18.8**	−26.8**	−20.9**	−13.5**	**10.4** **	−33.9**	−27.7**	−2.2	−7.1*	5.9	−31.4**
	Heterosis over PMS17B	**17.3** **	**15.7** **	−10.2*	−0.1	**7.5** *	−8.0*	−0.3	−12.6**	−14.3**	3.7	**10.3** **	−22.4**	−18.6**	**17.4** **	3.0	1.4	−20.7**
	Heterosis over CH 1or CH2	7.9	6.4	−17.3**	−8.1	**8.4** *	−7.2*	0.4	−11.9**	−13.6**	4.6	**11.3** **	−21.7**	−17.9**	**18.4** **	3.9	2.3	−20.5**
	Heterosis over recurrent parent	1.9	0.4	−22.0**	−13.2**	−14.2**	−26.6**	−20.5**	−30.3**	−31.6**	−17.2**	−11.9**	−38.0**	−35.1**	−6.29*	−17.8**	−19.0**	−36.5**
1000 grain weight (g)	Mean of test hybrid	25.5	24.9	27.1	25.0	26.3	25.0	24.9	26.4	26.9	26.1	24.7	27.1	25.4	28.3	25.1	23.9	24.8
	Heterosis over IL	**17.5** **	**7.17** *	**9.3** **	**6.8** *	**7.2** **	**6.8** *	**6.3** *	−1.8	−0.1	0.9	**8.4** **	−4.7	1.6	**12.4** **	−0.2	1.9	−4.5
	Heterosis over PMS17B	**15.1** **	**12.3** **	**22.2** **	**12.8** **	**17.5** **	**11.9** **	**11.4** **	**17.8** **	**20.1** **	**16.6** **	**10.5** **	**20.9** **	**13.5** **	**26.5** **	**12.1** **	**6.7** *	**10.9** **
	Heterosis over CH 1or CH2	**9.4** **	**6.8** *	**16.1** **	7.2*	**9.2** **	3.9	3.5	**9.4** **	**11.6** **	**8.3** **	2.6	**12.3** **	5.4	**17.5** **	4.1	−0.8	3.1
	Heterosis over recurrent parent	5.2	2.6	**11.6** **	3.1	−0.8	−5.6*	−6.0*	−0.6	1.3	−1.6	−6.8*	**1.9**	−4.3	**6.7** *	−5.4*	−9.9**	−6.4*
Grain yield (t/ha)	Mean of test hybrid	8.99	8.76	6.13	5.75	9.59	7.15	7.84	7.27	6.11	9.42	9.64	5.59	6.43	9.82	8.85	8.21	5.53
	Heterosis over IL	**30.3** **	**74.3** **	−5.3	−11.0	**23.6** *	−10.2	−6.4	**−19.1** *	−34.5**	**7.8**	**26.4** **	−36.7**	−19.4*	**51.6** **	5.6	**24.4** *	−33.1**
	Heterosis over PMS17B	**18.7** *	**15.6** *	−19.0*	−24.0**	**15.6**	−13.8	−5.4	−12.3	−26.2**	13.6	16.3	−32.6**	−22.4*	**18.4** *	6.6	−1.0	−33.2**
	Heterosis over CH 1or CH2	**33.3** **	**29.8** **	−9.1	−14.7	**39.6** **	4.0	14.1	5.7	−11.0	**37.1** **	**40.3** **	−18.6	−6.3	**43.0** **	**28.7** **	**19.4**	−19.4
	Heterosis over recurrent parent	**12.1**	9.2	−23.5**	−28.2**	**19.9** *	−10.6	−1.9	−9.1	−23.5**	**17.8** *	**20.5** *	−30.1**	−19.5*	**22.8** *	**10.6**	2.6	−30.7**

*, **significant at *P≤0.05* and *P≤0.01*, respectively.

CH1 = PMS17A/Pusa44; CH2 = PMS17A/PR114;

∧ILH developed from Pusa44 derived IL.

∼ ILH developed from PR114 derived IL;

a details of the hybrids are presented in table 1.

### Marker analysis and generation of graphical genotypes

**Figure 4:**
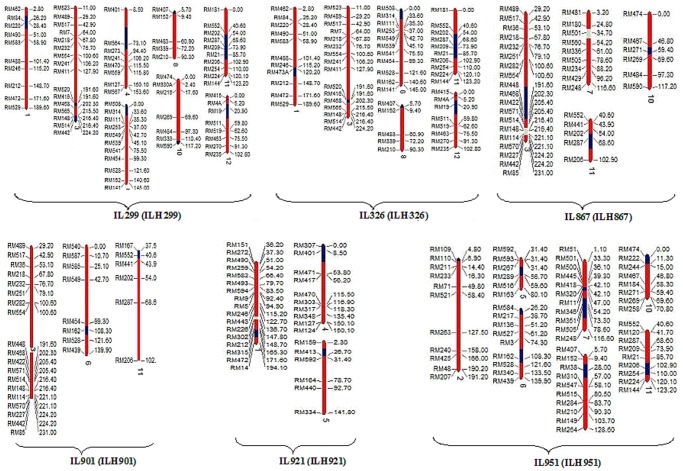
Graphical genotypes of six alien introgression lines generated after analyzing these with polymorphic SSR markers. Regions in blue are homozygous alien segments and gray are heterozygous alien segments introgressed from *O. rufipogon* in IL299, IL326, IL867 and IL901 and *O. nivara* in IL901 and IL951. Numbers on the right of linkage group are the cM distances as per Temnykh et al [32]. Numbers at the bottom are the chromosomes. Details of the ILHs in parentheses are presented in Table 1.

Introgression lines of six promising hybrids, viz., IL299, IL326, IL867, IL901, IL921 and IL951, which exhibited significant heterosis over their respective check hybrids, were used for the identification of alien segments responsible for enhanced performance of yield-related traits. A total of 200 SSR markers spanning all 12 chromosomes were used for characterizing each introgression line. Introgression lines, viz., IL299, IL326, IL867 and IL901, were in BC_2_F_6_ generation, whereas IL921 and IL951 were in BC_2_F_8_ generation. Introgression lines IL299 and IL326, which shared a common parentage, had 16 and 10 marker introgressions, (7.7% and 5.2%, respectively) from *O. rufipogon*. Introgression lines IL867 and IL901, which also shared a common parentage, had 3.4% and 2.5% genome introgressions from *O. rufipogon*. In introgression line IL921, only five markers (2.2%) showed introgressions from *O. nivara*, whereas line IL951 showed 12 marker introgressions (6.7%) from *O. nivara*. Details of introgressions in six introgression lines are presented in Table S3. Depending upon the introgression pattern of six introgression lines, graphical genotypes for each line were generated (Fig. 4).

### Associating yield and yield components with alien introgressed regions

Yield increase in hybrids, viz., ILH299, ILH326, ILH867, ILH901, ILH921 and ILH951, was caused by increase in panicle length, spikelets per panicle, grains per panicle and 1000-grain weight. Hybrids ILH299, ILH326, ILH867 and ILH901 showed increased 1000-grain weight over their respective introgression lines IL299, IL326, IL867 and IL901. Comparison of IL299, IL326, IL867 and IL901 revealed common introgressed regions on chromosomes 3 and 11. All the ILs had common introgressions around 191–202 cM region on chromosome 3 and 40–102 cM region on chromosome 11 (Fig. 4). Likewise, IL921 and IL951 derived from crosses with *O. nivara* had common introgressions around 31 cM region on chromosome 5. Introgression line IL299 had *O. rufipogon* allele at RM514, RM148 and RM442, whereas IL326 had RM416 on chromosome 3. IL867 carried *O. rufipogon* alleles at RM448, RM514, RM570, RM148 and RM114, whereas IL901 also had introgression for marker RM448 (Fig. 4). In the present study, ILH951 showed higher magnitude of heterosis for 1000-grain weight than IL951 and the checks. Introgression line IL951 had *O. nivara* alleles at RM222 (11.3 cM) on chromosome 10 and RM254 (110.0 cM) on chromosome11 (Fig. 4; Fig. S3). *O. rufipogon* and *O. nivara* alleles at these marker loci could be associated with increased 1000-grain weight in introgression line hybrids. ILH299 and ILH326 showed improvement in spikelets per panicle, grains per panicle and spikelet fertility over IL299 and IL326, respectively. Both the introgression lines had common introgression at marker loci RM508 (0.0 cM) on chromosome 6, RM407 (5.7 cM) on chromosome 8, RM202 (54.0 cM) to RM21 (85.0 cM) on chromosome 11 and RM19 (20.9 cM) on chromosome 12 (Fig. 4; Fig. S2). These introgression lines showed introgression of *O. rufipogon* chromatin at RM84, RM220 and RM473A on chromosome 1 and at RM21 on chromosome 11 (Fig. 4). ILH921 recorded higher yield than IL921, PMS17A/PR114 and parents because of increased panicle length, spikelet fertility, spikelets per panicle and grains per panicle. Molecular characterization of IL921 revealed introgressions of *O. nivara* chromatin at RM302 (147.8 cM) and RM472 (171.6 cM) on chromosome 1 and RM307 (0.0 cM) and RM401 (8.5 cM) on chromosome 4. ILH951 was the highest yielding among all hybrids because of highest spikelets per panicle, grains per panicle, spikelet fertility and 1000-grain weight. Parental introgression line (IL951) of this hybrid had introgression from *O. nivara* in seven chromosomes. Introgression was observed for RM109 (4.8 cM) on chromosome 1, RM289 (56.7 cM) on chromosome 5, RM584 (20.2 cM) and RM 351 (73.3 cM) to RM505 (78.6 cM) on chromosome 7, RM38 (28.0 cM) on chromosome 8, RM474 (0.0 cM) to RM222 (11.5 cM) on chromosome 10, and RM206 (102.9 cM) to RM254 (110.0 cM) on chromosome 11 (Fig. 4). The observed improvement in yield-contributing traits in this hybrid could be attributable to introgressions from *O. nivara.* In addition to yield components, days to 50% flowering and plant height decides the suitability of a hybrid for production. Introgression lines IL867 and IL901 were earlier than recurrent parent PR114, but their hybrids ILH867 and ILH901 took more days to mature than parental introgression lines. *O. rufipogon* alleles at marker locus RM287 on chromosome 11 in IL867 and IL901 might show increased effect on days to 50% flowering in their hybrids. Introgression line IL921 showed a significant increase in plant height over recurrent parent PR114, but its hybrid ILH921 had reduced plant height over IL921. *O. nivara* alleles at RM472 on chromosome 1 and RM307 on chromosome 4 could be responsible for increased plant height in IL921, but when these alleles are present in heterozygous condition in ILH921, there was reduction in plant height. Similarly, ILH951 showed a significant reduction in days to 50% flowering and days to maturity relative to IL951 and PMS17A/PR114.

## Discussion

Though rice has a wide genetic base, the germplasm with which breeders are working, in general, has limited variability, which probably is the major bottleneck in improving productivity. Because breeders, in general, are not currently using unadapted germplasm in their breeding programmes, pre-breeding must be strengthened to widen the genetic base of the working germplasm. Incorporation of ‘hidden genes’ in improved rice germplasm, through development and utilization of alien introgression lines for productivity enhancement, has emerged as a good breeding strategy [4]. The common wild rice species, *O. rufipogon* and *O. nivara*, are known as the ancestors of cultivated rice and have been proven to be a valuable gene pool for rice genetic improvement [35]. Sun et al. [36] studied the genetic diversity of *O. rufipogon* and *O. sativa* from more than 10 Asian countries and found that the genetic diversity of *O. rufipogon* was much higher than that of cultivated rice. A large number of alleles present in *O. rufipogon* could not be found in cultivated rice. Utilizing these hidden genes for the genetic enrichment of rice cultivars/hybrids could bring a revolutionary change in productivity improvement.

Introgression lines (ILs) serve as a valuable tool for genetic studies and genome analysis. A common accession of *O. rufipogon* (IRGC105491) was used in the development of alien ILs in most of the previous studies [14]–[16], [19], [37]–[39]. In the present study, out of 17 ILs used for generating F_1_ hybrids, six were derived by crossing two indica rice cultivars (Pusa44 and PR114) with two different accessions each of *O. rufipogon* (IRGC100219 and IRGC104433) and *O. nivara* (IRGC100593 and CR100142A). These were used for identification of alien segments responsible for enhanced magnitude of heterosis for yield and its component traits. In these ILs, 37 markers confirmed the presence of 32 alien segments dispersed across the genome. Sixteen alien segments, associated with 19 markers, have been located in the same or similar regions where QTL for yield and its component traits were mapped in previous studies [14]–[16], [20], [23], [37], [40]–[45] (Table 4). ILHs developed from these alien ILs, carrying introgressions from ‘A’ genome donor species, demonstrated the scope of utilization of alien introgressions in heterosis breeding. Results from two years of evaluation revealed that hybrids having introgressions from *O. rufipogon* (ILH299, ILH326, ILH867and ILH901) and *O. nivara* (ILH921 and ILH951) in their genome, showed significant heterosis for grain yield, panicle length, spikelets per panicle, grains per panicle and 1000-grain weight over introgression lines, check hybrids and hybrids generated by crossing the cms line and the recurrent parent. Superiority of introgression lines over recurrent parents have been reported by several workers, but the studies on heterosis involving alien introgression lines are quite limited. Xiao et al. [14] identified QTL for panicle length (*pl1.1, pl1.2, pl2.1, pl4.1, pl8.1, pl9.1*& *pl9.1*), spikelets per panicle (*spp1.1, spp1.2 & spp9.1*), grains per panicle (*gpp1.1, gpp8.2 & gpp12.1*), percent seed set (*pss2.1& pss4.1*), and 1000-grain weight (*gw4.1, gw8.1, gw9.1, gw11.1 & gw12.1*) from *O. rufipogon* in interspecific BC_2_ testcross population. *O. rufipogon* allele at two QTL, namely, *yld1.1* and *yld2.1*, was associated with 18 and 17 per cent grain yield increment, respectively. These two QTL were later transferred to the elite restorer lines Minghui 63, 93-11 and Ce64-7 through marker assisted selection and their functions were confirmed by significant yield improvement in rice hybrids [45]. In another study, involving F_1_ hybrids between a cross of *O. rufipogon*-derived ILs and recurrent parent Guichao2, 30 heterotic loci associated with six yield-related traits were reported by Luo et al. [20]. In tomato, Gur and Zamir [46] successfully increased fruit yield using ILs carrying a pyramid of three independent yield-promoting genomic regions introduced from wild species *Solanum pennellii*. Yield of hybrids developed using these pyramided ILs was more than 50% higher than that of a leading commercial variety. This study demonstrated the merit of ‘exotic alleles’ in improving crop productivity.

**Table 4:** Correspondence of reported introgressions from *O. rufipogon* and *O. nivara* with the already reported quantitative trait locus/loci.

Putative traits	ILs	Donor species	Introgression reported in chromosome	Marker associated with introgressed regions	Marker associated with QTL(s) and the species as reported in previous studies	Reference
Panicle length (cm)	IL921	*O. nivara*	1	**RM472**	**RM472** (*pl1*) *O. rufipogon*, RM315 (*pl1.1*) *O. rufipogon*, RM315 (*pl1.1*) *O. rufipogon*	[40], [16], [37]
Spikelets per panicle	IL299	*O. rufipogon*	1	**RM220**	RG532 (*spp1.2*) *O. rufipogon*, RM246 (*spp1.2*) *O. rufipogon*,**RM220** (*spp1*) *O. glumaepatula*	[14], [40], [41]
	IL326	*O. rufipogon*	1	RM473A	RM237 (*spp1.2*) *O. sativa* cv. Jefferson	[16]
	IL921	*O. nivara*	4, 1, 5	**RM307**, RM302, RM413	**RM307** (*hsp4*) *O. rufipogon*, RZ730 (*spp1.1*) *O. rufipogon*, RM194 (*snp5.1*) *O. rufipogon*	[20], [15], [42]
Grains per panicle	IL299	*O. rufipogon*	1	**RM220, RM84**	**RM220** (*gpl1.1*) *O. rufipogon*, **RM220** (*ggp1*) *O. glumaepatula*, **RM84** (*qGPL1*) *O. rufipogon*	[37], [41], [43]
	IL951	*O. nivara*	7, 10	**RM11, RM222**	**RM11** (*qFG7*) *O. rufipogon*, **RM222** (*hgp10*) *O. rufipogon*, C153A-**RM222** (*gp10*) *O. sativa*	[43],
	IL921	*O. nivara*	5	RM413	RM194 (*gn5.1*) *O. rufipogon*	[42]
Spikelet fertility (%)	IL921	*O. nivara*	1, 4	RM302, RM472, **RM307**	RM212 (*pss1.1*) *O. rufipogon*, RM315 (*sf1.1*) *O. rufipogon*, RM315 (*pss1.1*) *O. sativa* cv. Jefferson, **RM307** (*hspp4*) *O. rufipogon*	[37], [42], [16], [20]
	IL951	*O. nivara*	7, 5	**RM11, RM289**	**RM11** (*qss7*) *O. rufipogon*, **RM289** (*hssp5*) *O. rufipogon*	[43], [20]
1000 grain weight (g)	IL299	*O. rufipogon*	3, 11	RM442, **RM21**	RM227 (*gw3.1*) *O. rufipogon*, **RM21** (*hgw11*) *O. rufipogon*, RG2-**RM21** (*gw11b*) *O. sativa*	[15], [20], [44]
	IL323	*O. rufipogon*	11	**RM21**	**RM21** (*hgw11*) *O. rufipogon*	[20]
	IL867	*O. rufipogon*	3	RM514, RM570, RM180, RM114	RM227 (*gw3.1*) *O. rufipogon*	[16]
	IL951	*O. nivara*	10, 11	**RM222, RM254**	**RM222** (*hgw10*) *O. rufipogon*, C153A-**RM222** (*gw10*) *O. sativa*, G89-G1084 *(gw10) O. sativa*, **RM254** (*gw11.2*) *O. rufipogon*	[20], [44], [45], [15]
Grain Yield (t/ha)	IL299	*O. rufipogon*	1	**RM220**	**RM220** (*yld1.1*) *O. rufipogon*, **RM220** (*gyp1*) *O. glumaepatula*, **RM220** *O. glumaepatula*	[37], [41], [23]
	IL951	*O. nivara*	8, 2	**RM38**, RM109	**RM38** (*yld8.3*) *O. rufipogon*, RM236 (*hyp2a*) *O. rufipogon*	[42], [20]

Markers in bold are common regions associated with yield and yield components in various studies.

Most of the markers associated with the alien introgressions, in this study, are in correspondence with the already reported QTL for yield-contributing traits from *O. rufipogon* [14]–[16], [37], [40], [42]–[43] (Table 4). In accordance with the earlier reports, results of the present study also showed the positive association of alien segments with the yield-contributing traits and their utilization in hybrid development. Two experimental hybrids, viz., ILH921 (PMS17A/IL921) and ILH951 (PMS17A/IL951), developed by using PR114-derived ILs, carried *O. nivara* chromosomal segment in their genome. ILH921 showed increased yield over parent IL921 and check hybrid through yield-contributing traits. IL921 had *O. nivara* introgressions around markers RM302, RM472 in chromosome 1 and around marker RM307 in chromosome 4, which are same as where *O. rufipogon* alleles have been observed to confer positive effect on panicle length, spikelets per panicle and spikelet fertility in previous studies [14], [16], [20], [37], [40], [42] (Table 4). ILH951 was highest yielding among all the hybrids. Pollen parent of this hybrid, IL951, had introgressions from *O. nivara* in nine regions on seven chromosomes. Marker loci RM11, RM222, RM254, RM289 and RM109 on chromosomes 7, 10, 11, 5 and 2, respectively, exhibited presence of *O. nivara* chromatin. The same markers were reported to be linked to QTL transferred from *O. rufipogon* for grains per panicle, spikelet fertility, 1000-grain weight and yield per plant [15], [20], [42], [43] (Table 4). Markers responsible for improvement in yield traits through incorporation of genomic regions from *O. nivara* in the present study were also reported to be linked with QTL derived from *O. rufipogon*. Though these two species have distinct phenotypes because of their different life cycles and breeding behavior in their natural habitats, because of their evolutionary path they share some common/conserved genomic regions for yield component traits, as also observed in this study. *O. rufipogon* alleles seem to be effective across recipient genotypes and evaluation sites and *O. nivara* alleles showed synteny with the alleles of *O. rufipogon.* This synteny between genomic regions derived from both the alien species is well demonstrated by SSRs. Occurrence of intermediate populations between *O. nivara* and *O. rufipogon* types in some habitats and introgressions among these wild species and between cultivated and wild species [47]–[49] may be the reason for such homology. The complete information on the extent of introgression between these two wild species is still an enigma [50]–[53].

Pollen fertility of hybrids is important for realization of superior yields. Higher pollen fertility (>10%) in ILH299, ILH326, ILH901, ILH921 and ILH951 than that of check hybrids indicates the role of alien species-derived modifier genes present in ILs.

Introgression lines, viz., IL299, IL326, IL867, IL921 and IL951, did not show any improvement in grains per panicle, 1000-grain weight and grain yield when compared with recurrent parents, but their hybrids showed significant improvement over check hybrids and recurrent parents. Alien segments in heterozygous state showed an increasing effect for these traits (overdominance). It seems that in heterozygous condition, the recipient complement is conserved and the minor negative effects associated with alien segments are masked. Introgression in this set of ILs has been recorded in several chromosomal regions and some of these are large in size. These QTL, thus, need to be mapped for their precise transfer into other elite parental lines, as the superiority of the alien segment in heterozygous state can be readily exploited in hybrid rice breeding. The strength of utilization of related wild species in improving level of heterosis is well demonstrated through this experiment.

## Supporting Information

Figure S1 **(a–f): Frequency distributions of 318 ILHs for yield and yield contributing traits.**
(EPS)

Figure S2 **Ethidium bromide stained DNA amplification profile of parents and introgression lines using microsatellite markers.** Lanes are: M = 100 bp ladder, 1 = Pusa44, 2 = *O. rufipogon* (Acc. IR104852), 3 and 4 = introgression lines (IL299 and IL326), C = negative control.
(EPS)

Figure S3 **Ethidium bromide stained DNA amplification profile of parents and introgression lines using microsatellite markers.** Ethidium bromide stained DNA amplification profile of parents and introgression lines using microsatellite markers. Lanes are: M = 100 bp ladder, 1 = PR114, 2 = *O. nivara* (Acc. IR100142A), 3 = introgression line (IL951), C = negative control.
(EPS)

Table S1 **Mean square values for different agronomic traits based on ANOVA of field trials of introgression line hybrids.**
(DOC)

Table S2 **Mean values for some important agronomic traits and extent of heterosis (%) over parents, check hybrids and commercial checks in 17 hybrids.**
(DOC)

Table S3 **Proportion of microsatellite markers that observed introgression from** ***O. rufipogon*** **(IL299) and** ***O. nivara*** **(IL951).**
(DOC)
