# Supplementary material for: Yield-Enhancing Heterotic QTL Transferred from Wild Species to Cultivated Rice Oryza sativa L
Source: PLoS One. 2014 Jun 20;9(6):e96939. doi: 10.1371/journal.pone.0096939 (PMC4064972; doi:10.1371/journal.pone.0096939)
Supplement: Table S1 — Mean square values for different agronomic traits based on ANOVA of field trials of introgression line hybrids. (DOC) [file pone.0096939.s004.doc]

**Table S1: Mean square values for different agronomic traits based on ANOVA of field trials of introgression line hybrids.**

| Traits | Mean Squares | | | | | |
| --- | --- | --- | --- | --- | --- | --- |
|  | Experiment (2008)  Simple lattice design | | Experiment I (2009)  RBD | | Experiment II (2009)  RBD | |
|  | Replications | Genotypes | Replications | Genotypes | Replications | Genotypes |
| Days to 50% flowering | 2.47 | 51.24** | 5.64* | 129.08** | 1.16 | 93.04** |
| Days to maturity | 13.34** | 44.79** | 0.17 | 118.86** | 2.38 | 90.68** |
| Plant height | 0.67 | 46.11** | 17.14 | 67.96** | 5.11 | 134.99** |
| Productive tillers per plant | 41.30** | 1.52** | 1.80** | 0.15 | 0.50 | 0.55 |
| Panicle length | 14.70** | 2.60** | 0.67 | 6.30** | 0.21 | 7.80** |
| Spikelets per panicle | 1071.84 | 1208.41** | 887.44 | 2120.10* | 155.55 | 2343.29** |
| Grains per panicle | 592.83 | 5259.34** | 57.44 | 2471.05** | 337.56* | 937.37** |
| Spikelet fertility | 0.65 | 1159.44** | 29.14 | 138.32** | 22.28 | 296.52** |
| Pollen fertility | - | - | 10.97 | 201.08** | 179.70** | 198.25** |
| 1000 Grain weight | 2.65* | 5.20** | 1.25 | 5.93** | 0.74 | 7.13** |
| Yield (qt/ha) | 22.43 | 962.93** | 6.86 | 951.42** | 97.21 | 594.03** |

*, ** significant at *P < 0.05* and *P < 0.01*, respectively
