# Supplementary material for: Yield-Enhancing Heterotic QTL Transferred from Wild Species to Cultivated Rice Oryza sativa L
Source: PLoS One. 2014 Jun 20;9(6):e96939. doi: 10.1371/journal.pone.0096939 (PMC4064972; doi:10.1371/journal.pone.0096939)
Supplement: Table S2 — Mean values for some important agronomic traits and extent of heterosis (%) over parents, check hybrids and commercial checks in 17 hybrids. (DOC) [file pone.0096939.s005.doc]

**Table S2: Mean values for some important agronomic traits and** extent of heterosis (%) over parents, check hybrids and recurrent parents in 17 hybrids.

| Traits | Parameters | ILHs | | | | | | | | | | | | | | | | |
| --- | --- | --- | --- | --- | --- | --- | --- | --- | --- | --- | --- | --- | --- | --- | --- | --- | --- | --- |
|  |  | **ILH**  **299**^a | **ILH**  **326**^ | **ILH**  **411**^ | **ILH**  **435**^ | **ILH**  **867** ˜ | **ILH**  **873** ˜ | **ILH**  **894** ˜ | **ILH**  **896** ˜ | **ILH**  **900** ˜ | **ILH**  **901** ˜ | **ILH**  **921** ˜ | **ILH**  **924** ˜ | **ILH**  **929** ˜ | **ILH**  **951** ˜ | **ILH**  **1011** ˜ | **ILH**  **1459** ˜ | **ILH**  **1433** ˜ |
| Days to 50% flowering | Mean of test hybrid | **112.0** | **112.0** | **113.3** | **115.6** | **110.6** | **112.3** | **112.3** | **112.0** | **111.0** | **111.0** | **110.3** | **112.6** | **117.0** | **111.3** | **108.3** | **113.6** | **116.6** |
|  | Heterosis over IL | **-7.9**** | **-6.1**** | **-6.5**** | **-5.9**** | **2.7**** | **1.8*** | **2.1**** | **-0.5** | **2.7**** | **2.1**** | **-2.0**** | **-1.1** | **-7.6**** | **-9.2**** | **-5.8**** | **-10.0**** | **2.6**** |
|  | Heterosis over PMS17B | **-3.4**** | **-3.4**** | **-2.3**** | **-0.9** | **-4.3**** | **-2.8**** | **-2.8**** | **-3.1**** | **-4.0**** | **-4.0**** | **-4.6**** | **-2.5**** | **1.1** | **-3.7**** | **-6.3**** | **-1.7*** | **0.8** |
|  | Heterosis over CH 1or CH2 | **-4.0**** | **-4.0**** | **-2.8**** | **-0.8** | **-2.9**** | **-1.4*** | **-1.4*** | **-1.7*** | **-2.6**** | **-2.6**** | **-3.2**** | **-1.1** | **2.6**** | **-2.3**** | **-4.9**** | **-0.2** | **2.3**** |
|  | Heterosis over Recurrent parent | **-6.9**** | **-6.9**** | **-5.8**** | **-3.8**** | **-8.5**** | **-7.1**** | **-7.1**** | **-7.4**** | **-8.2**** | **-8.2**** | **-8.8**** | **-6.8**** | **-3.3**** | **-7.9**** | **-10.4**** | **-6.0**** | **-3.5**** |
|  | Mean of test hybrid | **0.9** | **0.9** | **2.1*** | **4.2**** | **-0.9** | **0.6** | **0.6** | **0.3** | **-0.6** | **-0.6** | **-1.1** | **0.9** | **4.7**** | **-0.3** | **-2.9**** | **1.7*** | **4.4**** |
| Plant height (cm) | Heterosis over IL | **104.1** | **104.4** | **103.2** | **95.1** | **116.6** | **104.5** | **104.6** | **103.2** | **105.4** | **109.4** | **114.9** | **103.6** | **105.6** | **103.4** | **105.4** | **101.2** | **101.4** |
|  | Heterosis over PMS17B | **2.7** | **-1.2** | **-1.5** | **7.0**** | **-4.7*** | **1.4** | **-9.7**** | **5.0** | **-3.5** | **4.3** | **-6.0**** | **0.9** | **-2.4** | **-0.3** | **6.5*** | **12.2**** | **-6.3*** |
|  | Heterosis over CH 1or CH2 | **2.7** | **3.0** | **1.9** | **-6.1**** | **13.9**** | **2.1** | **2.2** | **0.9** | **3.0** | **6.9*** | **12.3**** | **1.2** | **3.2** | **1.0** | **3.0** | **-1.1** | **-0.9** |
|  | Heterosis over Recurrent parent | **1.6** | **1.8** | **0.7** | **-7.1**** | **12.3**** | **0.7** | **0.8** | **-0.5** | **1.5** | **5.4*** | **10.7**** | **-0.1** | **1.8** | **-0.3** | **1.5** | **-2.5** | **-2.3** |
|  | Mean of test hybrid | **-1.9** | **-1.6** | **-2.7** | **-10.4**** | **13.8**** | **2.0** | **2.2** | **0.8** | **2.9** | **6.90*** | **12.2**** | **1.1** | **3.1** | **0.9** | **2.9** | **-1.1** | **-0.9** |
|  | Heterosis over IL | **5.1*** | **5.3*** | **4.2** | **-3.9** | **16.6**** | **4.5** | **4.6** | **3.2** | **5.4*** | **9.4**** | **14.9**** | **3.6** | **5.6*** | **3.4** | **5.4*** | **1.2** | **1.4** |
| Spikelets per panicle | Heterosis over PMS17B | **207.7** | **208.3** | **201.3** | **218.8** | **220.4** | **209.8** | **206.7** | **194.9** | **200.1** | **222.7** | **224.1** | **204.0** | **193.9** | **241.9** | **184.8** | **212.3** | **240.1** |
|  | Heterosis over CH 1or CH2 | **20.2** | **44.2**** | **11.3** | **20.3** | **21.3**** | **44.6**** | **18.5**** | **27.8**** | **25.0**** | **48.6**** | **24.3**** | **40.3**** | **16.1**** | **43.2**** | **25.6**** | **12.8**** | **49.8**** |
|  | Heterosis over Recurrent parent | **27.8** | **28.1** | **23.8** | **34.6*** | **1.3** | **-3.5** | **-4.9** | **-10.3**** | **-8.0*** | **2.4** | **3.0** | **-6.1** | **-10.8**** | **11.2**** | **-15.0**** | **-2.3** | **10.3**** |
|  | Mean of test hybrid | **31.1*** | **31.4*** | **27.0** | **38.1*** | **7.7*** | **2.5** | **1.0** | **-4.7** | **-2.2** | **8.8*** | **9.4*** | **-0.3** | **-5.2** | **18.1**** | **-9.7*** | **3.7** | **17.2**** |
|  | Heterosis over IL | **34.7*** | **35.1*** | **30.5*** | **41.9**** | **19.6**** | **13.9**** | **12.2**** | **5.8** | **8.6*** | **20.9**** | **21.6**** | **10.7*** | **5.3** | **31.3**** | **0.3** | **15.2**** | **30.3**** |
|  | Heterosis over PMS17B | **43.6**** | **44.0**** | **39.1*** | **51.2**** | **40.8**** | **34.0**** | **32.0**** | **24.5**** | **27.8**** | **42.2**** | **43.1**** | **30.3**** | **23.8**** | **54.5**** | **18.0**** | **35.6**** | **53.3**** |
| Pollen fertility (%) | Heterosis over CH 1or CH2 | **76.2** | **78.3** | **60.2** | **63.2** | **77.9** | **67.4** | **65.7** | **65.6** | **55.2** | **71.7** | **82.5** | **57.7** | **67.1** | **74.2** | **85.2** | **72.1** | **58.8** |
|  | Heterosis over Recurrent parent | **26.2*** | **3.4** | **-9.4** | **10.9** | **9.0** | **-9.0** | **-12.5*** | **-6.4** | **-20.6**** | **-4.5** | **19.3**** | **-26.7**** | **0.1** | **40.5**** | **28.2**** | **27.5**** | **-13.6*** |
|  | Mean of test hybrid | **10.8** | **14.0** | **-12.2** | **-8.0** | **9.3** | **-5.3** | **-7.7** | **-7.8** | **-22.4**** | **0.6** | **15.9*** | **-18.9**** | **-5.7** | **4.2** | **19.6**** | **1.2** | **-17.4**** |
|  | Heterosis over IL | **12.6** | **15.8** | **-10.8** | **-6.5** | **10.7** | **-4.2** | **-6.6** | **-6.7** | **-21.4**** | **1.8** | **17.3**** | **-18.0**** | **-4.6** | **5.4** | **21.0**** | **2.5** | **-16.4*** |
|  | Heterosis over PMS17B | **6.6** | **9.7** | **-15.5** | **-11.5** | **4.6** | **-9.4** | **-11.7** | **-11.8*** | **-25.7**** | **-3.7** | **10.8** | **-22.5**** | **-9.8** | **-0.3** | **14.4*** | **-3.1** | **-21.0**** |
|  | Heterosis over CH 1or CH2 | **-3.9** | **-1.2** | **-24.0**** | **-20.3*** | **-3.1** | **-16.2**** | **-18.3**** | **-18.4**** | **-31.3**** | **-10.9*** | **2.5** | **-28.3**** | **-16.6**** | **-7.7** | **5.8** | **-10.3** | **-26.9**** |

*, ** significant at *P < 0.05* and *P < 0.01*, respectively. CH1: PMS17A / Pusa44; CH2: PMS17A / PR114

^: ILH developed from Pusa44 derived IL. ˜: ILH developed from PR114 derived IL
