# Supplementary material for: Yield-Enhancing Heterotic QTL Transferred from Wild Species to Cultivated Rice Oryza sativa L
Source: PLoS One. 2014 Jun 20;9(6):e96939. doi: 10.1371/journal.pone.0096939 (PMC4064972; doi:10.1371/journal.pone.0096939)
Supplement: Table S3 — Proportion of microsatellite markers that observed introgression from O. rufipogon (IL299) and O. nivara (IL951). (DOC) [file pone.0096939.s006.doc]

**Table S3: Proportion of microsatellite markers that observed introgression from *O. rufipogon* and *O. nivara*** in six ILs.

| **Chromosome** | **Marker tested** | | | | | | **Polymorphic markers** | | | | | | **Marker showing introgression** | | | | | | **% Introgression** | | | | | |
| --- | --- | --- | --- | --- | --- | --- | --- | --- | --- | --- | --- | --- | --- | --- | --- | --- | --- | --- | --- | --- | --- | --- | --- | --- |
|  | IL 299 | IL 326 | IL 867 | IL 901 | IL 921 | IL 951 | IL 299 | IL 326 | IL 867 | IL 901 | IL 921 | IL 951 | IL 299 | lL 326 | IL 867 | IL 901 | IL 921 | IL 951 | IL 299 | IL 326 | IL 867 | IL 901 | IL 921 | IL 951 |
| **1** | 24 | 24 | 24 | 24 | 26 | 23 | 10 | 11 | 7 | 7 | 17 | 9 | 3 | 1 | 0 | 0 | 2 | 0 | 12.50 | 4.17 | 0.00 | 0.00 | 7.69 | 0.00 |
| **2** | 21 | 21 | 26 | 26 | 24 | 25 | 6 | 6 | 16 | 16 | 10 | 11 | 0 | 0 | 0 | 0 | 0 | 1 | 0.00 | 0.00 | 0.00 | 0.00 | 0.00 | 4.00 |
| **3** | 24 | 24 | 29 | 29 | 22 | 22 | 16 | 16 | 16 | 16 | 11 | 8 | 4 | 1 | 5 | 2 | 0 | 0 | 16.67 | 4.17 | 17.24 | 6.89 | 0.00 | 0.00 |
| **4** | 15 | 15 | 14 | 14 | 16 | 16 | 8 | 8 | 8 | 8 | 10 | 7 | 1 | 0 | 0 | 0 | 2 | 0 | 6.67 | 0.00 | 0.00 | 0.00 | 12.5 | 0.00 |
| **5** | 18 | 18 | 14 | 14 | 15 | 14 | 7 | 7 | 9 | 9 | 6 | 6 | 0 | 1 | 0 | 0 | 1 | 1 | 0.00 | 5.56 | 0.00 | 0.00 | 6.67 | 7.14 |
| **6** | 16 | 16 | 14 | 14 | 19 | 15 | 11 | 11 | 8 | 8 | 12 | 9 | 1 | 0 | 0 | 2 | 0 | 2 | 6.25 | 0.00 | 0.00 | 14.28 | 0.00 | 13.33 |
| **7** | 14 | 14 | 15 | 15 | 12 | 17 | 8 | 8 | 9 | 9 | 4 | 11 | 0 | 0 | 1 | 0 | 0 | 3 | 0.00 | 0.00 | 6.67 | 0.00 | 0.00 | 17.65 |
| **8** | 13 | 13 | 15 | 15 | 14 | 17 | 5 | 5 | 6 | 6 | 3 | 10 | 1 | 1 | 0 | 0 | 0 | 1 | 7.69 | 7.69 | 0.00 | 0.00 | 0.00 | 5.88 |
| **9** | 14 | 14 | 14 | 14 | 17 | 16 | 5 | 5 | 5 | 5 | 11 | 9 | 0 | 0 | 0 | 0 | 0 | 0 | 0.00 | 0.00 | 0.00 | 0.00 | 0.00 | 0.00 |
| **10** | 12 | 12 | 12 | 12 | 11 | 11 | 7 | 7 | 6 | 6 | 4 | 8 | 1 | 0 | 1 | 0 | 0 | 2 | 8.33 | 0.00 | 8.33 | 0.00 | 0.00 | 18.18 |
| **11** | 15 | 15 | 11 | 11 | 12 | 14 | 10 | 10 | 5 | 5 | 4 | 9 | 4 | 4 | 1 | 1 | 0 | 2 | 26.67 | 26.67 | 9.09 | 9.09 | 0.00 | 14.29 |
| **12** | 14 | 14 | 12 | 12 | 12 | 10 | 9 | 9 | 7 | 7 | 5 | 3 | 2 | 2 | 0 | 0 | 0 | 0 | 14.28 | 14.29 | 0.00 | 0.00 | 0.00 | 0.00 |
| **Total** | 200 | 200 | 200 | 200 | 200 | 200 | 102 | 103 | 102 | 102 | 97 | 100 | 16 | 10 | 8 | 5 | 5 | 12 | 7.66 | 5.21 | 3.44 | 2.52 | 2.24 | 6.71 |
